# Two New Monoterpene Glycosides from Qing Shan Lu Shui Tea with Inhibitory Effects on Leukocyte-Type 12-Lipoxygenase Activity

**DOI:** 10.3390/molecules18044257

**Published:** 2013-04-11

**Authors:** Hideyuki Ito, Akemi Otsuki, Hitomi Mori, Peng Li, Mai Kinoshita, Yuki Kawakami, Hideaki Tsuji, Ding Zhi Fang, Yoshitaka Takahashi

**Affiliations:** 1 Graduate School of Medicine, Dentistry and Pharmaceutical Sciences, Okayama University, 1-1-1 Tsushima-naka, Kita-ku, Okayama 700-8530, Japan; E-Mails: ph19139@s.okayama-u.ac.jp (H.M.); pnen3yvi@s.okayama-u.ac.jp (P.L.); 2 Faculty of Health and Welfare Sciences, Okayama Prefectural University, 111 Kuboki, Soja, Okayama 719-1197, Japan; E-Mails: hb24001a@fhw.oka-pu.ac.jp (A.O.); hb24002x@fhw.oka-pu.ac.jp (M.K.); kawaka@fhw.oka-pu.ac.jp (Y.K.); htsuji@fhw.oka-pu.ac.jp (H.T.); ytaka@fhw.oka-pu.ac.jp (Y.T.); 3 Department of Biochemistry and Molecular Biology, West China School of Preclinical and Forensic Medicine, Sichuan University, 17 Section 3, South Renmin Road, Chengdu 610041, China; E-Mail: dzfang@scu.edu.cn

**Keywords:** Qing Shan Lu Shui, *Ligustrum robustum*, liguroside, monoterpene glycoside, leukocyte-type 12-lipoxygenase, Chinese tea, catechin

## Abstract

We evaluated the inhibitory effect of 12 Chinese teas on leukocyte-type 12-lipoxygenase (LOX) activity. Tea catechins such as epigallocatechin gallate have been known to exhibit leukocyte-type 12-LOX inhibition. Qing Shan Lu Shui, which contains lower catechin levels than the other tested teas, suppressed leukocyte-type 12-LOX activity. To characterize the bioactive components of Qing Shan Lu Shui, leukocyte-type 12-LOX inhibitory activity–guided fractionation of the aqueous ethanol extract of the tea was performed, resulting in the isolation of two new monoterpene glycosides: liguroside A (**1**) and B (**2**). The structures of compounds **1** and **2** were characterized as (2*E*,5*E*)-7-hydroperoxy-3,7-dimethyl-2,5-octadienyl-*O*-(α-l-rhamnopyranosyl)-(1″→3′)-(4′″-*O*-*trans*-*p*-coumaroyl)-β-d-glucopyranoside and (2*E*,5*E*)-7-hydroperoxy-3,7-dimethyl-2,5-octa-dienyl-*O*-(α-l-rhamnopyranosyl)-(1″→3′)-(4′″-*O*-*cis*-*p*-coumaroyl)-β-d-glucopyranoside, respectively, based on spectral and chemical evidence. Ligurosides A (**1**) and B (**2**) showed inhibitory effects on leukocyte-type 12-LOX activity, with IC_50_ values of 1.7 and 0.7 μM, respectively.

## 1. Introduction

Oxidative modification of low-density lipoprotein is the first key step in the development of atherosclerosis, and the role of leukocyte-type 12-lipoxygenase (LOX) in this process has been established using leukocyte-type 12-LOX-knockout mice [1,2]. We previously reported that guava leaf extracts showed anti-atherogenic activity by inhibiting leukocyte-type 12-LOX activity [3]. Chinese teas derived from various kinds of plants are drunk for health benefits in parts of South China such as the province of Sichuan. Tea catechins such as epigallocatechin gallate and epicatechin gallate are known to inhibit several enzyme activities and to show inhibitory effects on inflammation and cardiovascular disease [4]. In the present study, we investigated the isolation and characterization of active constituents showing inhibitory effects on the leukocyte-type 12-LOX activity from Chinese teas.

## 2. Results and Discussion

**Figure 1:**
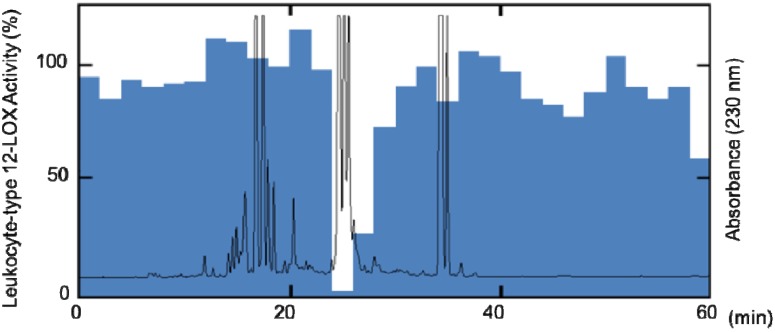
HPLC profile of Qing Shan Lu Chui extract and leukocyte-type 12-LOX activity of each fraction.

We evaluated the inhibitory effects of the 12 Chinese teas listed in Table 1 on leukocyte-type 12-LOX activity. Among the 12 Chinese tea extracts, Zhuya, Man Jian, Ganlu, Longjin Cha, Emei Mao Jian, Tie Guan Yin, and Qing Shan Lu Shui inhibited the conversion of arachidonic acid to 12-HPETE, which is catalyzed by leukocyte-type 12-LOX, with IC_50_ values of less than 20 µg/mL (Table 1). Tea catechins such as epigallocatechin gallate are known to inhibit leukocyte-type 12-LOX activity [5]. However, these inhibitors, except for those in Qing Shan Lu Shui, contained high amount of catechins (Table 1). Qing Shan Lu Shui [*Ligustrum robustum* (Roxb.) Blume] of the family Oleaceae contains quinic acid derivatives, flavonoids, and monoterpene glycosides, which have been reported to show antiviral and anti-inflammatory activities and inhibitory effects against the hemolysis of red blood cells induced by free radicals [6,7,8,9,10]. To characterize ingredients having inhibitory effects on leukocyte-type 12-LOX from Qing Shan Lu Shui, the dried tea leaves were extracted with 50% aqueous ethanol followed by ethyl acetate. Leukocyte-type 12-LOX inhibitory assay–guided fractionation of the 30% aqueous ethanol soluble portion of the ethyl acetate extract was carried out using reversed-phase HPLC, to yield fractions showing potent inhibitory activity at a retention time of 24–26 min (Figure 1). The active fractions were further purified by preparative HPLC to furnish two compounds, namely, ligurosides A and B.

**Table 1:** Inhibitory effect on leukocyte-type 12-LOX activity and catechin contents of 12 Chinese teas.

Chinese Teas	IC_50_ (μg/mL)	EGCg	Total Catechins
		(mg/g dried leaves)	
Zhuya	2.4	87.4	136.5
Man Jian	6.7	38.4	63.4
Ganlu	6.9	61.3	100.7
Longjin Cha	9.0	63.3	100.6
Emei Mao Jian	13.5	58.9	95.5
Tie Guan Yin	16.6	18.6	39.1
Qing Shan Lu Shui	19.3	0.6	0.7
Puer San Cha	20.0	20.8	41.3
Tou Cha	21.2	16.3	51.4
Tian Cha	26.8	<0.1	0.4
Hainan Kudig	38.1	<0.1	0.1
Qin Hua	>100	<0.1	0.1

EGCg: Epigallocatechin gallate.

**Figure 2:**
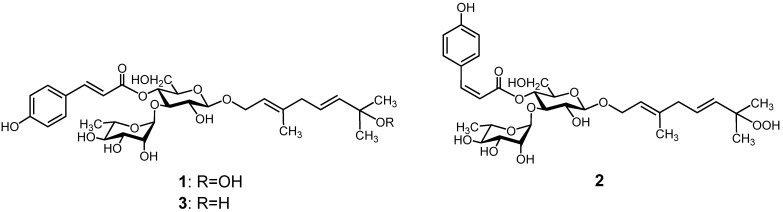
Structures of ligurosides A (**1**) and B (**2**), and kudingoside B (**3**).

**Figure 3:**
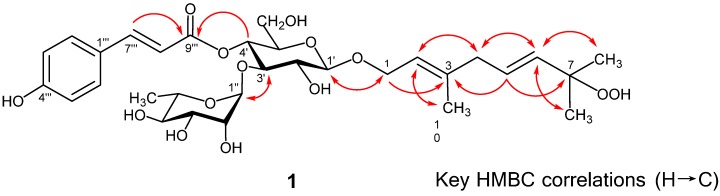
Key HMBC Correlations of liguroside A (**1**).

Liguroside A (**1**) was obtained as an off-white amorphous powder. Its molecular formula was determined as C_31_H_44_O_14_ on the basis of an HRESIMS peak at *m/z* 639.2665 [M−H]^−^ (calcd. for C_31_H_43_O_14_, 639.2658). The ^1^H-NMR spectrum of **1** showed AA′BB′-type protons at δ 7.55 and 6.89 (each 2H, d, *J* = 8.4 Hz) and *trans* olefin protons at δ 7.64 and 6.35 (each 1H, d, *J* = 15.6 Hz) in the aromatic proton region, corresponding to a *trans*-*p*-coumaroyl group. Proton signals due to a vinyl (δ 5.37), 2 mutually coupled vinyl (δ 5.67, 5.61), oxygenated methylene (δ 4.33, 4.21), a methylene (δ 2.75), a vinyl methyl (δ 1.67), and 2 tertiary methyl (δ 1.28) groups were observed in the aliphatic proton region. The presence of β-glucose and α-rhamnose residues was suggested by two anomeric proton signals at δ 4.40 (d, *J* = 7.2 Hz) and δ 5.29 (d, *J* = 1.8 Hz), and a secondary methyl proton signal at δ 1.09 (d, *J* = 6.6 Hz) (Table 2). The ^13^C-NMR and HSQC spectra for **1** displayed 31 carbon signals (listed in Table 2) due to 13 *sp*^2^ carbons, including an ester carbonyl carbon, 10 oxygenated methines, three methylenes, four methyls including a vinyl, and an oxygenated tertiary carbons, which were quite similar to those of *p*-coumaroyl, glucosyl, and rhamnosyl units in kudingoside B (**3**) [11] (Figure 2). The stereochemistry of sugars in **1** confirmed d-glucose and l-rhamnose on the basis of HPLC with an optical rotatory detector after acid hydrolysis of **1**. The locations of sugar and acyl units in **1** were established by an HMBC experiment (Figure 3).

**Table 2:** ^1^H- and ^13^C-NMR spectral data of ligurosides A (**1**) and B (**2**) in acetone-*d*_6_+D_2_O (δ_H_: 600 MHz; δ_C_: 150 MHz).

Position	1		2	
	δ_H_ (*J* in Hz)	δ_C_	δ_H_ (*J* in Hz)	δ_C_
Monoterpene				
1	4.21 dd (7.2, 12)	65.0	4.20 dd (7.2, 12)	65.9
	4.33 dd (6, 12)		4.32 dd (6, 12)	
2	5.37 dd (6, 7.2)	121.4	5.36 dd (6, 7.2)	122.2
3		138.7		139.6
4	2.75 d (6.6)	42.2	2.74 d (6.6)	43.1
5	5.61 dt (15.6, 6.6)	136.7	5.61 dt (15.6, 6.6)	137.6
6	5.67 d (15.6)	127.0	5.66 d (15.6)	127.8
7		80.6		81.4
8	1.28 s	24.1	1.27 s	24.9
9	1.28 s	24.1	1.27 s	24.9
10	1.67 s	15.6	1.65 s	16.5
Glucosyl				
1′	4.40 d (7.2)	101.4	4.38 d (8.4)	102.3
2′	3.42 dd (7.2, 9)	75.3	3.41 dd (8.4, 9)	76.1
3′	3.87 t (9)	78.7	3.83 t (9)	79.7
4′	4.89 t (9)	69.4	4.86 t (9)	70.1
5′	3.52 m	75.1	3.48 m	75.8
6′	3.59 m, 3.52 m	61.5	3.59 m, 3.50 m	62.4
Rhamnosyl				
1″	5.29 d (1.8)	100.9	5.28 d (1.8)	101.9
2″	3.86 m	71.1	3.87 m	71.9
3″	3.52 m	71.3	3.54 dd (3, 9.6)	72.2
4″	3.30 t (9)	72.7	3.32 t (9.6)	73.6
5″	3.62 dd (6.6, 9)	68.5	3.65 dd (6.6, 9.6)	69.4
6″	1.09 d (6.6)	17.6	1.14 d (6.6)	18.3
Coumaroyl				
1′″		126.1		127.2
2′″	7.55 d (8.4)	130.2	7.79 d (8.4)	134.1
3′″	6.89 d (8.4)	115.8	6.83 d (8.4)	115.7
4′″		159.9		159.8
5′″	6.89 d (8.4)	115.8	6.83 d (8.4)	115.7
6′″	7.55 d (8.4)	130.2	7.79 d (8.4)	134.1
7′″	7.64 d (15.6)	145.5	6.95 d (13.2)	145.9
8′″	6.35 d (15.6)	114.2	5.78 d (13.2)	115.9
9′″		166.2		166.0

In particular, the anomeric proton signal at δ 4.40 of glucose was correlated through three-bond coupling with C-1 resonance at δ 65.0 of a monoterpene unit. The signal of H-1 (δ 4.21 and 4.33) of the monoterpene unit was also correlated with the anomeric carbon (δ 101.4) of glucose. The other anomeric proton (rhamnose) at δ 5.29 displayed a correlation with the C-3′ carbon at δ 78.7 of glucose, and H-3′ at δ 3.87 of glucose was also correlated with the anomeric carbon at δ 100.9 of rhamnose. The H-4′ (δ 4.89) of glucose was correlated with H-7′′′ (δ 7.64) of *p*-coumaroyl unit through three-bond coupling with a common ester carbonyl carbon (δ 166.2). The L-rhamnopyranosyl-(1′′→3′)-(4′′′-*O*-*trans*-*p*-coumaroyl)-d-glucopyranosyl moiety was thus allocated to the C-1 position of the monoterpene system. The ^13^C resonances of the monoterpene unit of **1** were close to those of **3**, except for C-7 (δ 80.6 for **1** and δ 71.1 for **3**) and the tertiary methyl (C-8 and 9) (δ 24.1 for **1** and δ 30.0 for **3**) carbon signals [11] (Table 2). The remarkable downfield shift of C-7 and upfield shift of C-8 and C-9 resonances implied that the hydroxyl group of C-7 position in **3** for **1** is replaced by the hydroperoxy group [12,13]. The molecular formula of **1** calculated by HRESIMS analysis supports this suggestion. Furthermore, the reduction of **1** with triphenylphosphine yielded **3** [11], confirming the presence of a hydroperoxy group in the molecule. Consequently, the structure of liguroside A (**1**) was determined to be (2*E*,5*E*)-7-hydroperoxy-3,7-dimethyl-2,5-octadienyl-*O*-(α-l-rhamnopyranosyl)-(1′′→3′)-(4′′′-*O*-*trans*-*p*-coumaroyl)-β-d-glucopyranoside, as depicted in Figure 2.

Liguroside B (**2**), an off-white amorphous powder, exhibited a pseudomolecular ion peak at *m/z* 639.2641 [M−H]^−^ (calcd. for C_31_H_43_O_14_, 639.2658) in the HRESIMS, establishing its molecular formula as C_31_H_44_O_14_. The ^1^H-, ^13^C-NMR, MS, and UV spectral data of **2** were almost superposed on those of **1**, except for the coupling constant between H-7′′′ and H-8′′′ (*J* = 13.2 Hz) in the ^1^H-NMR spectrum. This indicates that the stereochemistry at C-7′′′ and C-8′′′ in **2** displays *cis* configuration. Liguroside B (**2**) was thus assigned to be a *cis* isomer at the *p*-coumaroyl moiety of **1**, namely, (2*E*,5*E*)-7-hydroperoxy-3,7-dimethyl-2,5-octadienyl-*O*-(α-l-rhamnopyranosyl)-(1′′→3′)-(4′′′-*O*-*cis*-*p*-coumaroyl)-β-d-glucopyranoside (Figure 2). The *cis* isomer of the *p*-coumaroyl moiety, which often exists in flavonoids, coumarins, and terpenoids, is well documented to be converted to its *trans* isomer under light conditions, such that *cis* and *trans* isomers occur as an equilibrium mixture [6,14,15]. In this study, we isolated each isomer as liguroside A (**1**, *trans* isomer) and liguroside B (**2**, *cis* isomer) under dark conditions; we also elucidated these structures. 

We evaluated the inhibitory effects of the new compounds isolated from Qing Shan Lu Chui on leukocyte-type 12-LOX activity. Ligurosides A (**1**) and B (**2**) exhibited significant inhibitory effects, with IC_50_ values of 1.7 and 0.7 μM, respectively. The inhibitory potency of liguroside A was comparable to that of quercetin (IC_50_ value of 1.6 μM) used as a positive control [3]. *p*-Coumaric acid, as a part of the catabolites believed to be produced from ligurosides by intestinal microflora after Qing Shan Lu Chui consumption showed no inhibition on leukocyte-type 12-LOX activity. These results suggested that the inhibitory effects of ligurosides A and B can be attributed to the monoterpene unit with the hydroperoxy group.

## 3. Experimental

### 3.1. General

Twelve Chinese teas (Zhuya, Man Jian, Ganlu, Longjin Cha, Emei Mao Jian, Tie Guan Yin, Qing Shan Lu Shui, Puer San Cha, Tou Cha, Tian Cha, Hainan Kudig, and Qin Hua) were purchased at a specialty tea market in Sichuan, China. Optical rotations were recorded on a Jasco DIP-1000 instrument. UV spectra were measured on a Jasco V-530 spectrometer. ^1^H- and ^13^C-NMR spectra were measured in acetone-*d*_6_ on a Varian NMR System 600 MHz (600 MHz for ^1^H-NMR and 150 MHz for ^13^C-NMR) instrument; chemical shifts are given in δ (ppm) values relative to that of the solvent (δ_H_: 2.04; δ_C_: 29.8) on a tetramethylsilane scale. The standard pulse sequences programmed for the instrument were used for each 2D-NMR experiment (^1^H-^1^H COSY, NOESY, HSQC, and HMBC). The *J*_CH_ value was set at 8 Hz in the HMBC experiment. Mass spectra were obtained on a Bruker MicrOTOF II spectrometer using ESI source in negative-ion mode. HPLC was performed on a Waters Alliance 2625 separations module (Milford, MA, USA). Reversed-phase HPLC was conducted on a 250 × 4.6 mm i.d. Inertsil ODS-3 column (GL Sciences, Inc., Tokyo, Japan) developed with 0.01% acetic acid (solvent A) and 0.01% acetic acid–methanol (solvent B) at 40 °C and a flow rate of 1.0 mL/min. Detection was effected at 200–700 nm. A gradient was applied as follows: the proportion of solvent B in the eluent increased from 30% to 100% (t = 50 min) and 100% (t = 60 min).

### 3.2. Quantification of Catechins in Extracts of Chinese Teas

Dried tea leaves (1 g) were extracted with 50% aqueous ethanol (20 mL) for 30 min. After filtration and evaporation *in vacuo* of the extract, the residue was re-dissolved with 50% aqueous ethanol (10 mg/mL), centrifuged (12,000 rpm, 4 °C, 5 min), filtered (0.45 μm), and injected into the HPLC system. Quantification of tea catechins [(+)-catechin, (−)-catechin, epicatechin, gallocatechin, gallocatechin gallate, epicatechin gallate, epigallocatechin, epigallocatechin gallate, and catechin gallate obtained from Wako Pure Chemical Industries, Ltd., Osaka, Japan] was performed on the basis of the absolute calibration curve method. HPLC was performed on a Waters Alliance 2695 separations module. Quantitative HPLC was conducted on a 250 × 4.6 mm i.d. Inertsil ODS-3 column (GL Sciences, Inc.) eluted with 0.05% phosphoric acid (solvent A) and 0.05% phosphoric acid–acetonitrile (solvent B) at 40 °C and a flow rate of 1.0 mL/min. Detection was effected at 280 nm. A gradient was applied as follows: the proportion of solvent B in the eluent increased from 5% to 10% (t = 15 min) and 45% (t = 40 min). The calibration curves were linear and had mean correlation coefficients of ≥0.99.

### 3.3. Leukocyte-type 12-LOX Inhibitory Assay

Porcine leucocyte-type 12-LOX was partially purified from J774A.1 cells overexpressing the enzyme [16]. The partially purified leukocyte-type 12-LOX was preincubated for 5 min at 30 °C with indicated concentrations of inhibitors in a standard 200-μL reaction mixture containing 100 mM Tris-HCl buffer (pH 7.4), and then incubated with 25 μM arachidonic acid (5 nmol/5 μL of ethanol solution) for 5 min at 30 °C. The products were reduced by the addition of glutathione peroxidase (0.1 U) and 5 mM glutathione, followed by incubation for a further 20 min. After acidification of the reaction mixture by the addition of 50 mM HCl, 0.5 nmol of 15-HEDE was added as an internal standard for LOX reaction. The products extracted with ice-cold diethyl ether were analyzed by reverse-phase HPLC, using a Waters Alliance system equipped with a COSMOSIL 5C_18_-MS-II column (5-μm particle, 250 × 4.6 mm i.d.; Nacalai, Kyoto, Japan) with a solvent system of methanol–water–acetic acid (80:20:0.01, v/v) at a flow rate of 1 mL/min, as previously described [17]. Absorption at 235 nm was continuously monitored, using a Waters 2489 UV/Visible detector. The products that co-chromatographed with authentic 12-HETE and 15-HETE in the leucocyte-type 12-LOX reaction were quantified by comparison of the areas of the peaks with that of an internal standard. Linear relationship was confirmed by calibration curves (five-point measurements) for conjugated dienes of produced HETEs, as well as internal standards. Protein concentration was determined using a bicinchoninic acid protein assay kit with bovine serum albumin as a standard.

### 3.4. Extraction and Isolation

The dried leaves of Qing Shan Lu Shui (1 g) were extracted with 50% aqueous ethanol (20 mL) for 30 min. After filtration and evaporation of the extract, the residue was partitioned between water (2 mL) and ethyl acetate (2 mL × 2). The organic layer was evaporated, and then, the resulting residue (110 mg) was re-dissolved with 30% aqueous ethanol (11 mL). The aqueous ethanol extract was purified by preparative HPLC to afford liguroside A (**1**) (retention time 31.5 min, 8.5 mg) and liguroside B (**2**) (retention time 37.6 min, 3.1 mg). HPLC was performed on a Waters Alliance 2695 separations module. Reversed-phase HPLC in an isocratic condition was conducted on a 250 × 4.6 mm i.d. Inertsil ODS-3 column (GL Sciences, Inc.) developed with methanol–water–acetic acid (47:53:0.01) at 40 °C and a flow rate of 1.0 mL/min. Detection was effected at 200–700 nm.

*Liguroside A* (**1**), An off-white amorphous powder; 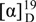 −81.2° (*c* 0.3, MeOH); UV λ_max_ (MeOH) nm (log ε): 316 (4.40); ^1^H- and ^13^C-NMR, see Table 2; HRESIMS *m/z* 639.2665 [M−H]^−^, calcd. for C_31_H_44_O_14_-H, 639.2658.

*Reduction of* **1** *with Triphenylphosphine*, A methanol solution (1 mL) of **1** (1 mg) was stirred for 12 h at ambient temperature with triphenylphosphine (Sigma-Aldrich, Tokyo, Japan) (1 mg). The reaction mixture was evaporated and purified by Bond Elut C_18_ cartridge column to yield **3** (0.3 mg) [10], HRESIMS *m/z* 623.2723 [M−H]^−^, C_31_H_44_O_13_-H, requires 623.2709.

*Acid Hydrolysis of* **1**. A solution of **1** (3 mg) in 1% H_2_SO_4_ (3 mL) was heated in a boiling water bath for 1 h. The reaction mixture was extracted with ethyl acetate. The ethyl acetate extract was analyzed by normal phase HPLC [250 × 4.6 mm i.d. YMC-Pack SIL-003 column (YMC Co., Ltd., Kyoto, Japan) with *n*-hexane–methanol–tetrahydrofuran–formic acid (55:33:11:1) containing oxalic acid (450 mg/L) at ambient temperature and a flow rate of 1.5 mL/min] to detect *p*-coumaric acid (2.9 min). The aqueous layer was neutralized with Diaion SA-20AP and evaporated. The residue was analyzed by HPLC equipped with an optical rotation detector (Jasco OR-2090, Jasco International Co., Tokyo, Japan), which demonstrated the retention time of the sugars to be identical with those of authentic d-glucose (9.3 min) and l-rhamnose (6.7 min). HPLC was performed on a Jasco Borwin system with a 250 × 4.6 mm i.d. TSK-gel Amide-80 column (Tosoh Co., Tokyo, Japan) developed with acetonitrile-water (82:18) at ambient temperature and at a flow rate of 1.0 mL/min.

*Liguroside B* (**2**), An off-white amorphous powder; 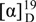 −88.8° (*c* 0.18, MeOH); UV λ_max_ (MeOH) nm (log ε): 315 (4.81); ^1^H- and ^13^C-NMR, see Table 2; HRESIMS *m/z* 639.2641 [M−H]^−^, calcd. for C_31_H_44_O_14_-H, 639.2658.

## 4. Conclusions

We have investigated the inhibitory effects of 12 commercial Chinese teas on leukocyte-type 12-LOX activity. Catechins such as epigallocatechin gallate have been known to inhibit leukocyte-type 12-LOX. Indeed, tested catechin-rich teas were found to attenuate the activity of the enzyme. However, Qing Shan Lu Shui, which contains lower levels of catechin among the tested teas, suppressed 12-LOX activity. Bioassay-guided fractionation of Qing Shan Lu Shui led to the isolation of two new monoterpene glycosides, named liguroside A (**1**) and B (**2**). Compounds **1** and **2** were determined to be (2*E*,5*E*)-7-hydroperoxy-3,7-dimethyl-2,5-octadienyl-*O*-(α-l-rhamnopyranosyl)-(1′′→3′)-(4′′′-*O*-*trans*-*p*-coumaroyl)-β-d-glucopyranoside and (2*E*,5*E*)-7-hydroperoxy-3,7-dimethyl-2,5-octadienyl-*O*-(α-l-rhamnopyranosyl)-(1′′→3′)-(4′′′-*O*-*cis*-*p*-coumaroyl)-β-d-glucopyranoside, respectively, on the basis of spectral and chemical evidence. Ligurosides A (**1**) and B (**2**) showed inhibitory effects on leukocyte-type 12-LOX activity, with IC_50_ values of 1.7 and 0.7 μM, respectively, while *p*-coumaric acid corresponding to the gut microbial metabolite of ligurosides showed no effect. These results implied that the inhibitory effect of ligurosides A and B on leukocyte-type 12-LOX activity is attributed to the monoterpene moiety having a hydroperoxy group. Although further details about the activity of ligurosides A and B are needed, our findings suggest that Qing Shan Lu Shui consumption might be beneficial in the prevention of atherosclerosis.
